# A non-destructive plant screening method for improving sample uniformity in horticultural crops based on a hydrogen peroxide fluorescent probe

**DOI:** 10.3389/fpls.2026.1767323

**Published:** 2026-03-09

**Authors:** Weisen Lan, Hongjie Chen, Bingying Zou, Qingyuan Yi, Xia Lin, Junrong Xu, Tianying Lei, Junwei Zhang, Xinyuan Chen, Peng Wang, Wenjin Yu

**Affiliations:** 1 College of Agriculture, Guangxi University, Nanning, China; 2 School of Energy and Building Environment, Guilin University of Aerospace Technology, Guilin, China; 3 Medical College, Guangxi University, Nanning, China; 4 Faculty of Pharmacy, Guangxi Health Science College, Nanning, China

**Keywords:** fluorescent probe, H_2_O_2_, nondestructive, plants, prescreening, semi-quantitative

## Abstract

**Introduction:**

Hydrogen peroxide (H_2_O_2_) functions as a key signaling molecule in plants responding to stress. Although numerous detection methods have been developed, simple and non-destructive techniques for the semi-quantitative monitoring of H_2_O_2_ in plant tissues remain scarce.

**Methods:**

In this study, we developed a "turn-on" fluorescent probe specifically designed to detect endogenous H_2_O_2_ in plant tissues, and conducted spectroscopic and in vivo toxicity tests. Furthermore, under experimentally controlled stress conditions, we utilized this probe to detect H_2_O_2_ levels in four distinct plant types exposed to salt, waterlogging, cadmium, and drought stresses. Additionally, H_2_O_2_ was detected in a grafting model under non-experimentally controlled stress conditions.

**Results:**

The results showed that the probe demonstrated excellent selectivity, a strong linear correlation (R2 = 0.9849), and a low detection limit of 0.6450 μmol/L. Importantly, it exhibits good biocompatibility with plant tissues and effectively minimizes detection errors caused by transient H_2_O_2_ fluctuations induced by environmental changes. Consequently, it provides more accurate and stress-reflective H_2_O_2_ measurements. Under experimentally controlled stress conditions, the changes in relative fluorescence intensity conformed to the typical response patterns observed when plants experience graded levels of stress. Notably, even under complex grafting conditions without imposed stress gradients, applying the probe to bottle gourd (Lagenaria siceraria) rootstocks with different graft compatibility produced fluorescence dynamics consistent with the typical H_2_O_2_ responses of compatible and incompatible rootstocks, and the distribution of relative fluorescence intensity within the population underscored the importance of prescreening plants for biological studies. Pearson correlation and Bland-Altman analyses confirmed good agreement between our method and the commercial assay kit.

**Discussion:**

These results demonstrate that the LWS probe enables H_2_O_2_ detection and, in combination with the IVIS in vivo imaging system, can screen individual plants differing in stress responses more effectively than other sensors. This non-destructive approach preserves the structural integrity of plant samples, enabling follow-up physiological, biochemical, and genomic analyses on the same specimens. This method provides a reliable prescreening platform for investigating plant stress responses at the biological level.

## 1 Introduction

With the intensification of global climate change and environmental pressures, plants are increasingly subjected to both biotic and abiotic stresses, such as drought, salinity, elevated temperatures, and pathogen infections. These stresses pose significant threats to plant growth, development, and yield. Therefore, gaining an in-depth understanding of the mechanisms underlying plant stress resistance is crucial for improving crop adaptability (Cotrina Cabello et al., 2023; Ahlawat et al., 2024). In recent years, omics technologies have provided powerful tools for studying plant stress resistance mechanisms. However, current omics sampling strategies primarily rely on fixed time points following stress treatment, overlooking the variability among individual plants (Shulaev et al., 2008; Muthamilarasan et al., 2019). Inter-individual variability in plants is a critical issue in biological research. Even within the same cultivar, under identical environmental conditions and uniform stress treatments, significant differences can arise in both response timing and intensity. Specifically, some individuals may not yet reach their maximum response at predefined sampling points, while others may have already passed their peak response. This temporal asynchrony introduces samples that are inconsistent with the targeted oxidative status, ultimately affecting the accuracy and reliability of omics data. (Cortijo et al., 2019; Schupp et al., 2019; Watahiki and Trewavas, 2019) Therefore, improving the uniformity of physiological states contributes to enhancing data quality and analytical accuracy. Furthermore, current research on plant stress resistance mechanisms typically involves the propagation of multiple plant generations, enabling the selection of individuals or varieties with varying resistance levels for further analysis. These plants exhibit differences in substances such as H_2_O_2_, plant hormones, antioxidants, and other related compounds (Asaeda et al., 2017; Liaqat et al., 2024). Conventional screening methods for disease-resistant plant varieties predominantly rely on the observation of typical symptom development induced by pathogens under field conditions. However, the monitoring and evaluation of disease symptoms throughout the entire growing season can require a long period of time, and visual assessments may not consistently and accurately reflect the resistance levels, as certain resistant plants may still exhibit mild symptoms at the sampling time points (Tibebu and Nuh, 2019; Jennings et al., 2024). Following stress exposure, physiological changes in plants occur earlier than the onset of visible symptoms. Quantitative measurements of these physiological parameters provide objective data, ensuring more reliable results compared to evaluations that depend on visible symptoms (Mutka and Bart, 2015). Consequently, physiological profiling of plant stress responses enables precise identification of resistant phenotypes, significantly accelerating the selection process in breeding programs aimed at developing stress-resilient crop varieties.

Elevation of reactive oxygen species (ROS) levels represents one of the most prominent physiological alterations in plants exposed to various biotic and abiotic stresses in natural environments. ROS encompass a group of redox-active molecules, primarily comprising H_2_O_2_, superoxide anion (·O_2_^-^), hydroxyl radical (·OH), and nitric oxide radical (NO·) (Hasanuzzaman et al., 2021). Under normal physiological conditions, ROS in plant cells are maintained at low levels through the regulatory action of the antioxidant system. Upon exposure to stress, ROS production rates are dramatically enhanced, exceeding the scavenging capacity of the antioxidant system and consequently leading to a marked elevation in ROS accumulation. Such ROS accumulation activates a series of signal transduction pathways, triggering plant defense responses and adaptive mechanisms (Farooq et al., 2019). H_2_O_2_ represents the most stable ROS, characterized by an extended half-life and superior diffusion capacity (Sharova et al., 2023; Henschel et al., 2024). This enables H_2_O_2_ to function not only as a local signaling molecule within cells but also to be transported over long distances through the apoplast and intercellular spaces, mediating intercellular signal transmission. As a result, plants are able to coordinate responses across different tissues and organs, thereby enhancing overall stress resistance (Slesak et al., 2007). As a signaling molecule, H_2_O_2_ can regulate the secretion of plant hormones in a dose-dependent manner, thereby influencing plant growth, development, and defense responses. Low concentrations of H_2_O_2_ typically promote plant growth and development, moderate concentrations can induce defense responses, while high concentrations may cause cellular damage or even cell death (Saxena et al., 2016; Nazir et al., 2020). Therefore, H_2_O_2_ serves as an indicator of the extent of plant damage and acts as a key determinant in the regulation of other plant hormones. Non-destructive assessment of H_2_O_2_ concentrations in plants can facilitate a better understanding of plant responses to stress and assist in the selection of more stress-tolerant individuals, thereby significantly enhancing breeding efficiency.

Traditional methods for detecting H_2_O_2_, such as chemiluminescence, electrochemical techniques, and chromatography, offer high sensitivity and accuracy but typically require destructive sampling of plant tissues. This limitation prevents the use of the same batch of samples for further studies, even when the response trends of plants under stress have been identified. Fluorescent proteins, as a non-invasive approach for detecting H_2_O_2_, can be expressed in living cells to emit fluorescence. However, their low transformation efficiency and unstable expression levels have restricted their widespread application in practical settings (Li et al., 2021; Mizuta, 2021). Small-molecule fluorescent probes have become currently among the most widely used non-invasive methods for detecting H_2_O_2_. These probes generate a fluorescent signal upon reaction with H_2_O_2_, enabling the monitoring of H_2_O_2_ concentration changes without causing destructive damage to the target being observed (Yang et al., 2023). Fluorescent probes for H_2_O_2_ detection commonly utilize boronate esters and their derivatives as recognition groups. Upon attack of the boron atom by H_2_O_2_, the boron-carbon bond is cleaved, releasing the corresponding phenolic derivative and altering the properties of the fluorescent probe, thereby enabling H_2_O_2_ detection. However, boronate esters are prone to hydrolysis in aqueous solutions, which can affect the synthesis and storage of the probes. Consequently, the preparation of fluorescent probes containing boronate groups requires researchers to possess extensive expertise and proficient synthetic skills, making such probes less accessible for agricultural and forestry scientists (Dhoun et al., 2017; Liang et al., 2021; Kirchner et al., 2024; Peng et al., 2024). Other fluorescent probes that employ acetyl, sulfonate, benzoyl, and similar groups as recognition moieties avoid the hydrolysis issues associated with boronate esters during synthesis and can be used to monitor oxidative stress changes in living organisms. However, these probes are predominantly utilized for the qualitative analysis of H_2_O_2_, making precise comparisons and statistical analyses difficult. This limitation restricts in-depth studies on the role of H_2_O_2_ and the biological responses it triggers (Wu et al., 2019; Yin et al., 2022; Li et al., 2023a, b; Wang et al., 2023). Although these probes have demonstrated excellent performance and some H_2_O_2_ fluorescent probes have been applied to plants, especially in imaging on plant root tips, the results are encouraging. However, these works lack simple and easily reproducible procedures and methods for the semi-quantitative detection of H_2_O_2_ in biological organisms while preserving intact plants. As a result, researchers in the fields of agriculture and forestry have shown little interest in these works, which has limited the application of fluorescent probe technology in the agricultural and forestry sectors.

**Figure 1:**
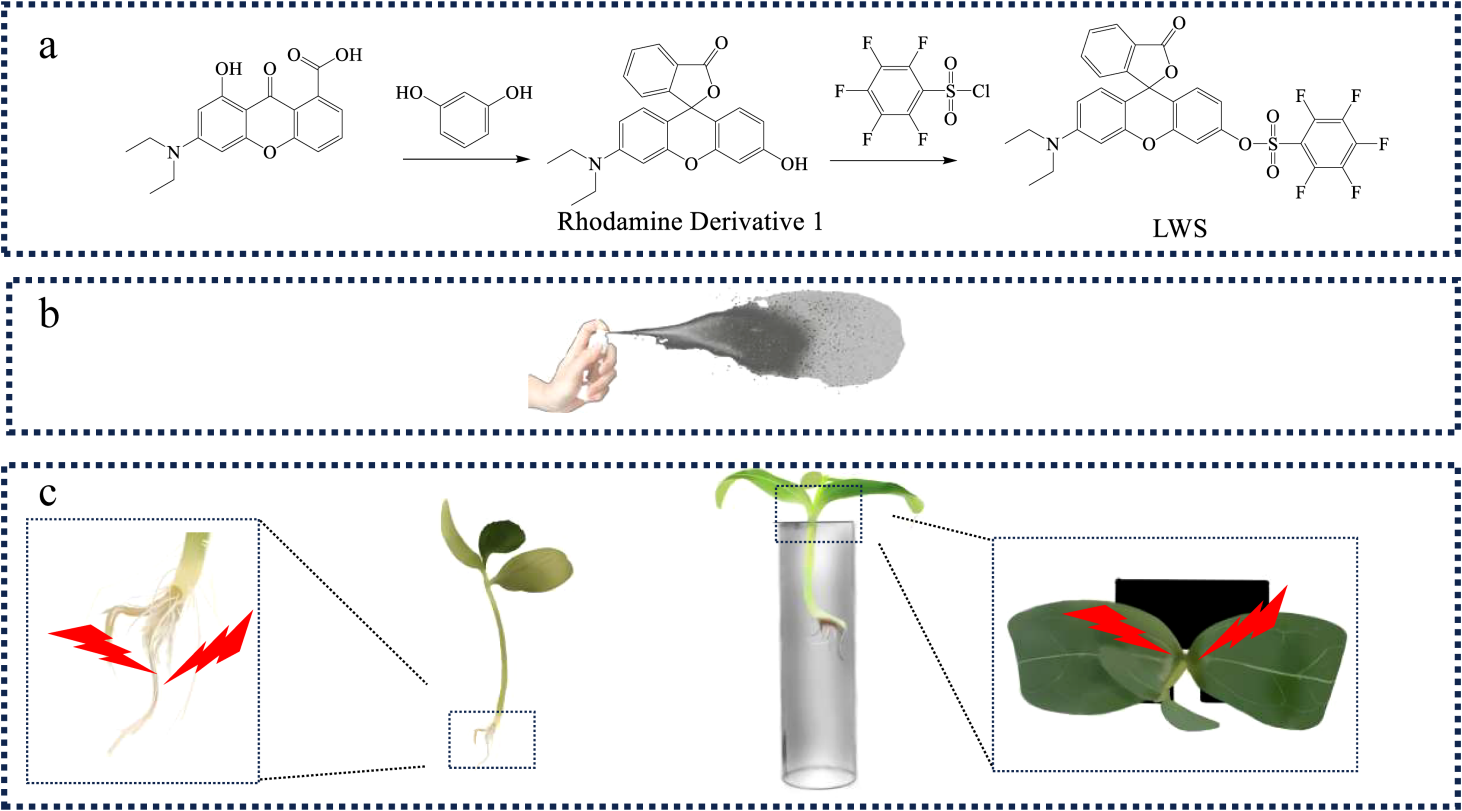
**(a)** Step 1: Synthesis of the fluorescent probe, **(b)** Step 2: Application of the fluorescent probe solution to plants using a small sprayer, **(c)** Step 3: Fluorescence imaging and photography.

In this study, a novel fluorescent probe for detecting H_2_O_2_ in plants was synthesized using a rhodamine derivative and pentafluorobenzenesulfonyl chloride as raw materials through a two-step chemical reaction (**Figure 1a**). Using this probe in combination with the IVIS Lumina LT *in vivo* imaging system, H_2_O_2_ levels in different parts of intact plants can be detected with only simple sample preparation, while preserving the integrity of the plant. Quantitative results reflecting relative fluorescence intensity changes induced by H_2_O_2_ can be obtained (**Figures 1b, c**). This new probe, together with the IVIS Lumina LT system, demonstrates promising potential for assisting researchers in the agricultural and forestry fields in screening plants exhibiting different degrees of damage.

## 2 Materials and methods

### 2.1 Experimental instruments and reagents

All analytical instruments were used as specified: nuclear magnetic resonance (NMR) spectrometer (AVANCE NEO, Bruker Corporation), high-resolution mass spectrometer (Xevo G2-XS Qtof, Waters Corporation), mass spectrometer (LTQ Orbitrap Elite, Thermo Fisher Scientific), *In vivo* plant imaging system (IVIS Lumina LT, PerkinElmer, USA), UV-Vis spectrophotometer (TU-1901, Persee), and fluorescence spectrophotometer (RF-5301 PC, Shimadzu Corporation). All chemicals and solvents were commercially available and used without further purification.

### 2.2 Synthesis of the compound

#### 2.2.1 Synthesis of rhodamine derivative 1

A mixture of 1.00 g of 2-[4-(diethylamino)-2-hydroxybenzoyl]benzoic acid and 0.35 g of resorcinol was added to a round-bottom flask and dissolved in 8 mL of trifluoroacetic acid (TFA). The reaction mixture was heated to 90 °C under stirring for 24 h. The solvent was subsequently removed under reduced pressure using a rotary evaporator, yielding a crude solid product. Purification via silica gel column chromatography with an ethyl acetate/petroleum ether eluent system (5:3, v/v) afforded a reddish-brown solid (1.13 g, yield: 90%).

^1^H NMR (600 MHz, DMSO-*d_6_*) δ 8.15 (d, J = 7.7 Hz, 1H), 7.85 (td, J = 7.5, 1.3 Hz, 1H), 7.78 (td, J = 7.6, 1.2 Hz, 1H), 7.41 (d, J = 7.5 Hz, 1H), 7.01 (s, 1H), 6.90 (dq, J = 28.3, 12.6, 9.2 Hz, 5H), 1.18 (t, J = 7.0 Hz, 6H).
^13^C NMR (151 MHz, DMSO-*d_6_*) δ 167.69, 159.21, 158.93, 158.66, 158.38, 155.05, 134.26, 131.10, 130.88, 130.77, 117.86, 115.93, 115.52, 102.60, 97.02, 45.58, 12.91.
MS: Calcd for [C24H21NO4]^+^ 387.1500, found [M + H]^+^ 388.3420.

#### 2.2.2 Synthesis of the fluorescent probe LWS

Rhodamine derivative 1 (1.00 g) was accurately weighed and dissolved in a stoichiometric volume of dichloromethane (DCM) in a round-bottom flask, which was then immersed in an ice-water bath. Under continuous stirring, triethylamine (0.39 g) was added dropwise. After 10 min, a dichloromethane solution (20 mL) containing pentafluorobenzenesulfonyl chloride (0.84 g) was slowly added to the reaction mixture via an addition funnel over 30 min. Following complete addition, the ice bath was removed, and the mixture was stirred at ambient temperature (25°C) for 12 h. The reaction solvent was evaporated under reduced pressure using a rotary evaporator to obtain the crude product. Purification through column chromatography with a dichloromethane/petroleum ether gradient (5:1, v/v) yielded the target probe LWS as a pink crystalline solid (1.02 g, 64% yield).

^1^H NMR (600 MHz, CDCl_3_) δ 8.05 (d, *J* = 7.5 Hz, 1H), 7.71 (td, *J* = 7.5, 1.3 Hz, 1H), 7.23-7.18 (m, 2H), 6.87 (dd, *J* = 8.7, 2.4 Hz, 1H), 6.82 (d, *J* = 8.8 Hz, 1H), 6.60 (d, *J* = 8.9 Hz, 1H), 6.51 (s, 1H), 6.46 - 6.40 (m, 1H), 3.40 (q, *J* = 7.1 Hz, 4H), 1.21 (t, *J* = 7.0 Hz, 6H).
^13^C NMR (151 MHz, CDCl_3_) δ 169.28, 152.64, 152.51, 152.48, 152.15, 151.96, 149.46, 142.78, 139.29, 135.91, 135.18, 135.12, 131.17, 130.07, 129.91, 128.87, 126.92, 125.14, 124.78, 124.02, 123.49, 119.78, 116.12, 115.91, 114.08, 110.20, 29.71, 29.66, 14.13, 12.42.
MS: Calcd for [C30H20F5NO6S]^+^ 617.0931, found [M + H]^+^ 618.1035.

The ^1^H NMR, ^13^C NMR spectra and mass spectra of the compounds are shown in support information.

### 2.3 Spectrophotometric measurement and selectivity analysis

A stock solution of probe LWS (10 mmol/L) was prepared by dissolving the appropriate quantity of LWS in dimethyl sulfoxide (DMSO). A 30% H_2_O_2_ solution was diluted with distilled water to obtain a 10 mmol/L H_2_O_2_ stock solution, which was freshly prepared prior to each experiment. The 10 mmol/L H_2_O_2_ stock solution was serially diluted to concentrations ranging from 0 to 100 µmol/L. These diluted H_2_O_2_ solutions were then mixed with the LWS probe solution (20 µmol/L in PBS: DMSO (1:1, v/v)) for subsequent analyses.

The pH of the probe LWS solution was adjusted using standardized hydrochloric acid (HCl) or sodium hydroxide (NaOH) solutions, covering a pH range of 3.0 to 11.0.

Selectivity experiments for plant hormones, ions, and various ROS were conducted. Plant hormones were dissolved in DMSO, while ions and other ROS were dissolved in distilled water. Fluorescence of LWS before and after the addition of analytes was monitored under an excitation wavelength of 522 nm. For other ROS, singlet oxygen (^1^O_2_, 50 mmol/L) was generated by adding H_2_O_2_ (500 mmol/L) to a sodium molybdate (50 mmol/L) solution. ·O_2_^-^ was produced by reacting 300 μmol/L xanthine with 10 mU/mL xanthine oxidase. ·OH (50 mmol/L) were generated by adding H_2_O_2_ (500 mmol/L) to a ferrous sulfate (50 mmol/L) solution. Nitric oxide (50 mmol/L) was prepared by dissolving 149 mg of sodium nitroprusside (SNP) in deionized water to a final volume of 10 mL. Tert-butyl hydroperoxide (TBHP, 50 mmol/L) solution was prepared by diluting 69 μL of 70% tert-butyl hydroperoxide with deionized water to 10 mL. During spectral measurements, the excitation wavelength was set at 522 nm, and the fluorescence emission spectra were recorded from 540 to 800 nm.

### 2.4 Toxicity experiment of the fluorescent probe on plants

Bottle gourd seeds were soaked in water at 55°C for 10 minutes, then transferred to fresh water and soaked overnight at room temperature. The seeds were subsequently germinated in a 32°C incubator. Once radicles emerged, the seeds were sown into 32-cell seedling trays. When the seedlings reached the two-leaf and one-heart stage, bottle gourd seedlings of similar size were selected for toxicity tests. The experimental groups included a control group (deionized water, CK) and treatment groups (10-100 µmol/L probe concentrations), with three replicates per group. After hydroponic culture for 72 hours, root length, stem length, stem diameter, fresh weight, and dry weight were measured.

### 2.5 Visualization imaging of H_2_O_2_ in plants

Seeds of Bottle gourd, Tomato(*Solanum Lycopersicum L.*), Pumpkin(*Cucurbita* spp.), and Melon(*Cucumis melo L.*) were raised into seedlings following the procedures described in Section 2.4. Littleleaf boxwood(*Buxus microphylla.*) and Orange(*Citrus reticulata.*) were obtained from the planting base.

After washing the root substrates from all plants, size and vigor-matched individuals were cultured in Hoagland nutrient solution until they acclimated to a hydroponic environment. Salt stress in Bottle gourd was imposed with 0, 50, or 100 mmol/L NaCl. Drought stress in Littleleaf boxwood was imposed with 0%, 10%, or 20% PEG6000. Cadmium stress in Orange was imposed with 0, 100, or 200 µmol/L CdCl_2_. The stress duration for these three treatments was 24 h. Tomato was subjected to waterlogging using a solution containing 100.1 mg/L KHCO_3_, 69.0 mg/L MgSO_4_, and 91.7 mg/L CaCl_2_, and H_2_O_2_ was measured at 0, 24, and 48 h after stress initiation. Each treatment had three replicates. At the preset stress time points, roots were rinsed three times with distilled water and then transferred to a solution containing the fluorescent probe for a 1 h incubation. Following incubation, the roots were rinsed three times with distilled water, and surface moisture was removed using absorbent paper. Fluorescence imaging was performed using the fluorescent mode of the IVIS Lumina LT *in vivo* imaging system. (excitation wavelength: 522 nm, emission wavelength: 552 nm). After imaging, to ensure the biological relevance of the fluorescence signal, an appropriate region of interest (ROI) was selected using Living Image software to calculate relative fluorescence intensity.

Bottle gourd cultivars H02 and H12, along with pumpkin NC03T, were used as rootstocks. When the seedlings reached the two-leaf and one-heart stage, grafting was performed using melon as the scion by the top-insertion method. H_2_O_2_ levels were monitored on days 1, 3, 5, and 7 after grafting, with three replicates per time point. A small sprayer was used to apply the fluorescent probe solution to the grafting site, and after 1 hour, imaging was performed using the IVIS Lumina LT *in vivo* imaging system. To ensure consistent imaging height, grafted seedlings were placed inside 12-cm-high glass tubes during imaging. Relative fluorescence intensity was quantified using the fluorescent mode of the IVIS Lumina LT by selecting a 0.3×0.4 cm ROI around the graft junction to calculate the total ROI values(excitation wavelength: 522 nm, emission wavelength: 552 nm), thereby ensuring the biological relevance of the fluorescence signal. In addition, hydrogen peroxide levels in grafted seedlings subjected to the same treatments were determined using a hydrogen peroxide assay kit (Solarbio, Cat: BC3590, Spectrophotometer, China) according to the manufacturer’s instructions.

All data were obtained from three biological replicates (10 seedlings per replicate) and are expressed as relative fluorescence intensity (photons per second, p/s). Statistical analysis was performed using one-way analysis of variance (ANOVA) with Duncan’s multiple range test for *post hoc* comparison of means, using SPSS software (version 27, IBM Corp.). Differences were considered statistically significant at p < 0.05. To eliminate differences in data scale between the two methods, the measurement data were normalized, and Bland–Altman analysis and Pearson’s correlation coefficient were then used to evaluate the agreement between the method proposed in this study and the commercial kit-based assay.

## 3 Results

### 3.1 Synthesis of the fluorescent probe LWS

The synthetic route of probe LWS is illustrated in **Figure 1a**. The synthesis begins with a cyclization reaction under acidic conditions between the starting materials 2-(4-diethylamino)-2-hydroxybenzoylbenzoic acid and resorcinol, yielding the rhodamine derivative 1. Under basic conditions, compound 1 is subsequently reacted with pentafluorobenzenesulfonyl chloride to afford the target probe LWS as a pink solid (64% yield). The structures of rhodamine derivative 1 and LWS were confirmed by ^1^H NMR, ^13^C NMR, and HRMS spectroscopy (Supporting Information).

### 3.2 Response of probe LWS to H_2_O_2_ *in vitro*

**Figure 2:**
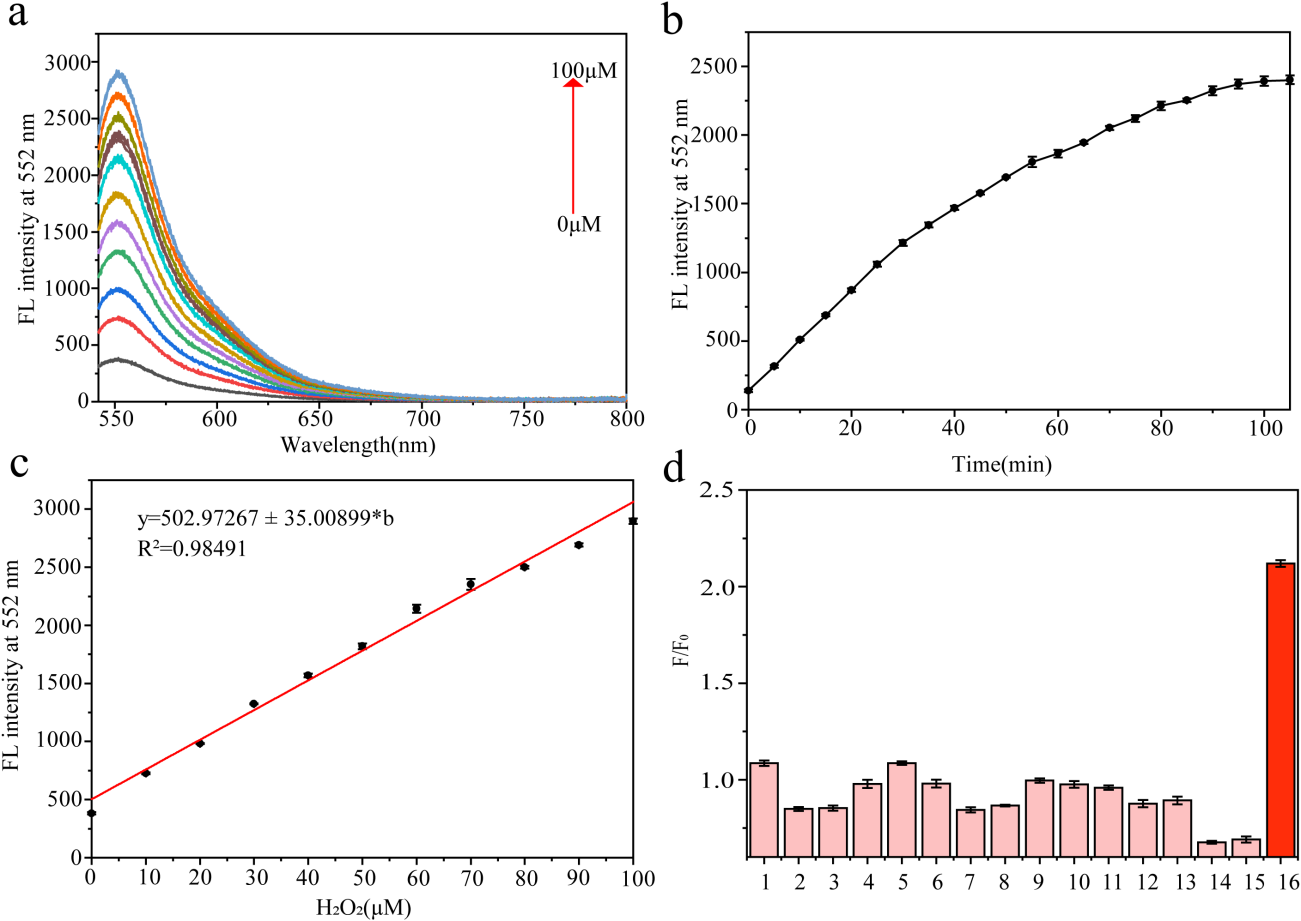
**(a)** Fluorescence spectrum of the LWS fluorescent probe with H_2_O_2_ (0-100 µmol/L), **(b)** Time dependence of the fluorescence probe on H_2_O_2_, **(c)** Linear relationship between fluorescence intensity at 552 nm and H_2_O_2_ concentration (0-100 µmol/L), **(d)** Fluorescence spectrum of the fluorescent probe in the presence of substances that may coexist with H_2_O_2_ in plants (1: ¹O_2_, 2: O_2_^-^, 3: ·OH, 4: NO·, 5: TBHP, 6: MeJA, 7: Abscisic acid, 8: Salicylic Acid, 9: Zeatin, 10: GA3, 11: IAA, 12: Ca^2+^, 13: Fe^2+^, 14: Fe^3+^, 15: Catalase, 16: H_2_O_2_).

The UV-visible absorption spectrum of the probe solution (**Supplementary Figure 1**) shows that probe LWS exhibits a characteristic absorption peak at 522 nm. As depicted in **Figure 2a**, the fluorescence spectrum of the probe solution was recorded using 522 nm-the wavelength of maximum absorption-as the excitation wavelength. Upon the addition of varying concentrations of H_2_O_2_, the fluorescence intensity at 552 nm increased significantly. To investigate the response speed of LWS toward H_2_O_2_, time-course fluorescence measurements were performed at various time points. As shown in **Figure 2b**, the fluorescence emission intensity stabilized after approximately 100 minutes upon the addition of H_2_O_2_. The pH within plant bodies generally ranges from 7.0 to 7.5, but depending on environmental conditions and different plant parts, the internal pH can vary from slightly acidic to slightly alkaline. To ensure that our fluorescent probe can react with H_2_O_2_ within plant bodies, we assessed the effect of different pH conditions on the probe’s performance. As shown in **Supplementary Figure 2**, the fluorescent probe was able to react with H_2_O_2_ and emit fluorescence at 552 nm within the pH range of 6.0 to 8.0. To ensure that the probe’s response aligns with the environmental and physiological pH conditions within plant bodies, we selected pH 7.4 for subsequent experiments (Felle, 1988; Yu et al., 2000) The response of probe LWS to varying concentrations of H_2_O_2_ is shown in **Figure 2c**. With increasing concentrations of H_2_O_2_, the fluorescence emission intensity increased accordingly. A good linear relationship was observed in the range of 0-100 µmol/L H_2_O_2_ (R^2^ = 0.9849), with a regression equation of y = 502.97267z ± 35.00899[H_2_O_2_]. The calculated limit of detection (LOD) was 0.645 µmol/L. The specificity of probe LWS for H_2_O_2_ was further examined, as shown in **Figure 2d**. Among all tested species, LWS exhibited the most pronounced fluorescence response toward H_2_O_2_. In contrast, no significant fluorescence enhancement was observed upon the addition of other ROS (^1^O_2_, ·O_2_^-^, ·OH, NO·, TBHP); plant hormones that may coexist with H_2_O_2_ in plant bodies (methyl jasmonate, abscisic acid, salicylic acid, zeatin, auxin, gibberellin); representative cations (Ga^2+^, Fe^2+^, Fe^3+^); or catalase.

**Figure 3:**
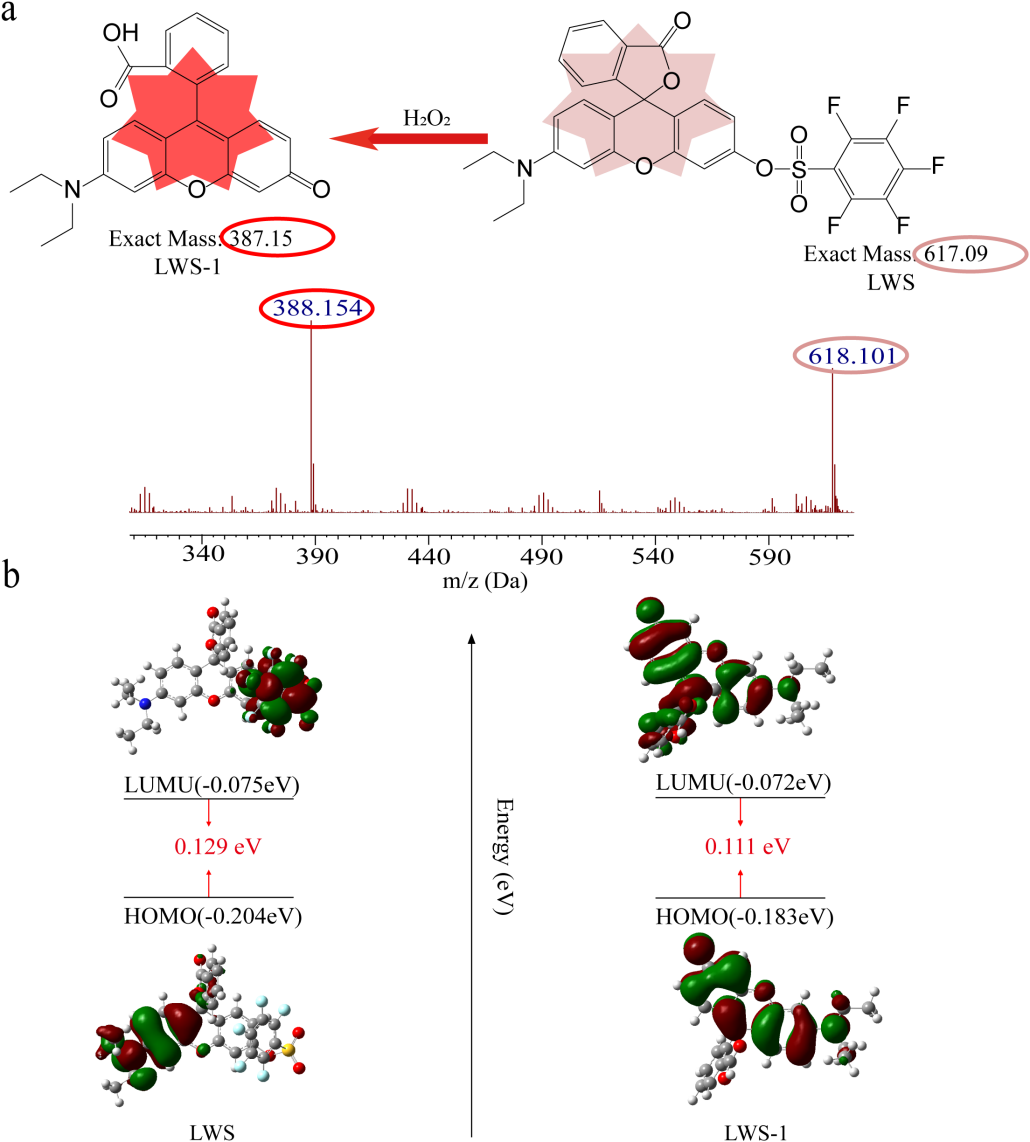
**(a)** Mass spectrum of the fluorescence probe LWS after reacting with the target H_2_O_2_, **(b)** DFT calculation of the frontier molecular orbital energy levels for LWS (left) and LWS-1 (right).

To gain deeper insight into the response mechanism of the fluorescent probe LWS toward H_2_O_2_, comparative analysis was performed on the reaction residues of LWS with H_2_O_2_ using high-resolution mass spectrometry (HRMS). As shown in **Figure 3a**, in addition to the molecular ion peak of LWS (m/z 618.101), a new peak was observed at m/z 388.154, consistent with the formation of the reaction product LWS-1. This response mechanism was further investigated using density functional theory (DFT) calculations carried out with the Gaussian 16 software package, employing the B3LYP/6-31G(d,p) basis set. As shown in **Figure 3b**, for the unreacted LWS probe, the π-electron cloud of the LUMO orbital was mainly localized on the pentafluorobenzenesulfonyl group. Upon activation by H_2_O_2_ and conversion to LWS-1, this group was cleaved, and the electron cloud became delocalized over the entire fluorophore. Moreover, the LUMO-HOMO energy gap of the activated product LWS-1 (0.111 eV) was smaller than that of the unreacted probe LWS (0.129 eV).

### 3.3 Response of Probe LWS to H_2_O_2_ *in vivo*

#### 3.3.1 Toxicity of the fluorescent probe on plants

**Figure 4:**
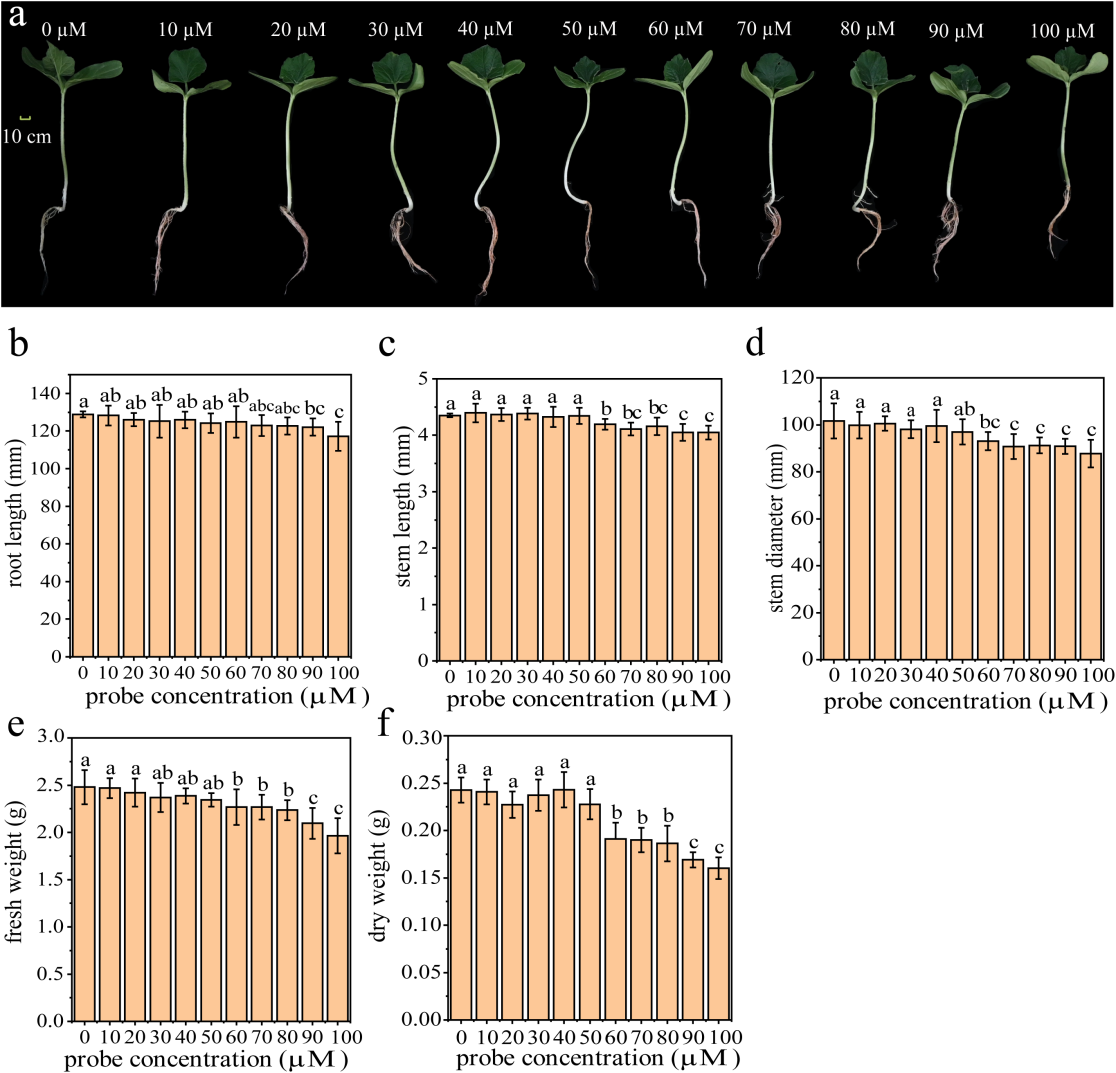
**(a)** Plant growth morphology, **(b)** Effect of 0-100 µmol/L fluorescent probe concentration on root length, **(c)** Effect of 0-100 µmol/L fluorescent probe concentration on stem length, **(d)** Effect of 0-100 µmol/L fluorescent probe concentration on stem diameter, **(e)** Effect of 0-100 µmol/L fluorescent probe concentration on fresh weight, **(f)** Effect of 0-100 µmol/L fluorescent probe concentration on dry weight.

Before investigating whether the fluorescent probe could be applied for *in vivo* imaging in plants, we conducted a phytotoxicity test on the fluorescent probe in living plants. As shown in **Figure 4a**, bottle gourd seedlings were treated with different concentrations of the fluorescent probe. Except for the seedlings treated with distilled water, whose roots retained their original color, those exposed to 10-100 µmol/L probe solutions had their roots in direct contact with the probe. During the three-day treatment, H_2_O_2_ produced by normal plant growth was continuously detected, and the reaction product of the fluorescent probe with H_2_O_2_ accumulated in the roots, resulting in the red coloration of the bottle gourd seedling root systems. To confirm that this color change does not affect the normal growth and development of plants, we measured the root length, stem length, stem diameter, fresh weight, and dry weight of bottle gourd seedlings (**Figures 4b–f**). Statistical analysis showed that at probe concentrations of 50 µmol/L or lower, neither the fluorescent probe nor its reaction product with H_2_O_2_ had a significant effect on the growth of either the aboveground or underground parts of the seedlings, nor did they significantly impact fresh or dry biomass. Therefore, to ensure maximal detection of H_2_O_2_ within plants without interfering with plant development, a probe concentration of 50 µmol/L was selected for subsequent applications in plant systems.

#### 3.3.2 Detection of in plant H_2_O_2_ induced by experimentally controlled stress gradients using the fluorescent probe LWS

**Figure 5:**
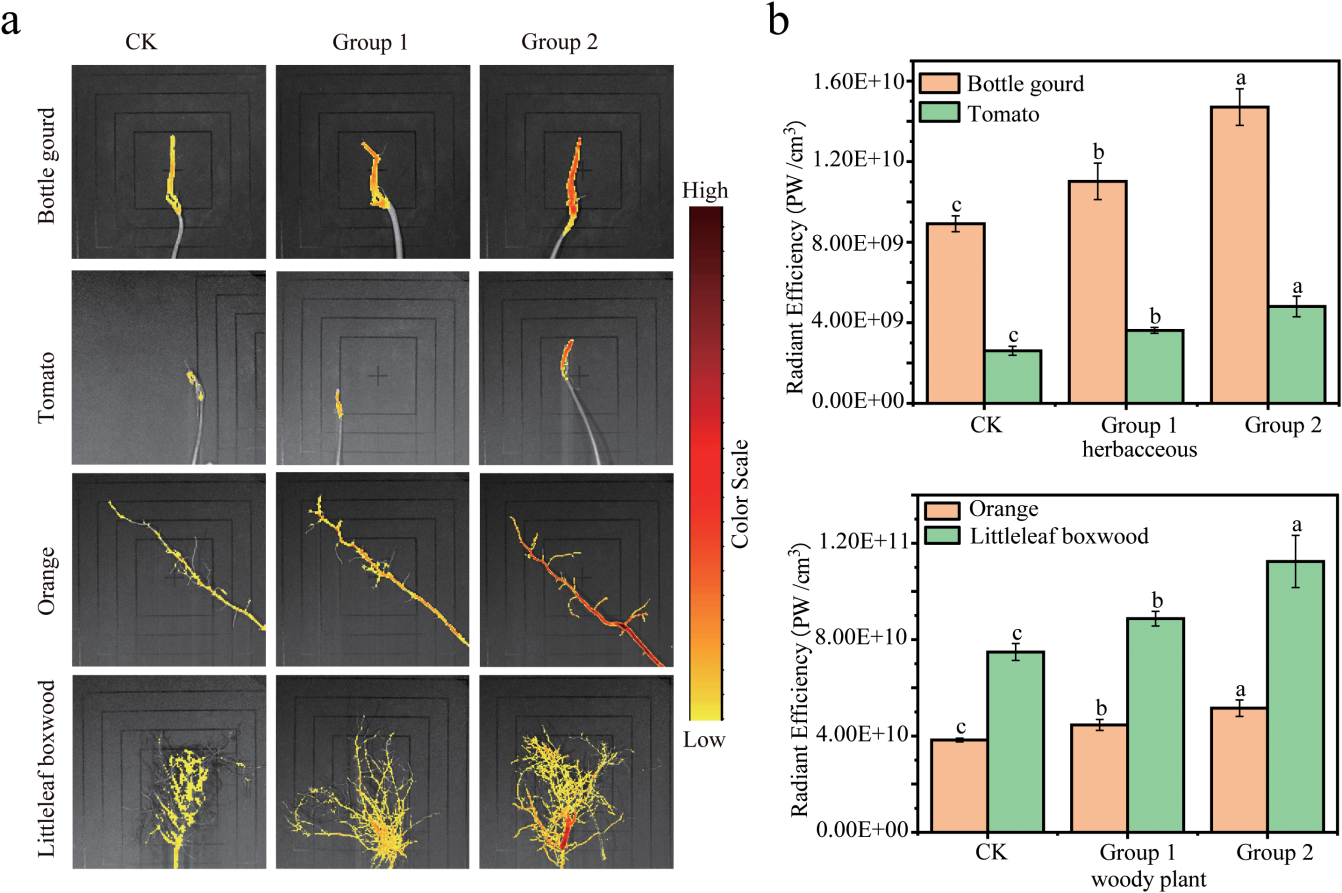
Fluorescence imaging of plant roots using the fluorescent probe LWS. **(a)** Bottle gourd under salt stress ck is the control, group 1 is 50 mmol/L NaCl, group 2 is 100 mmol/L NaCl, color scale from 1.00e8 µW/cm^2^ to 1.00e9 µW/cm^2^. Tomato under waterlogging stress ck is 0 h, group 1 is 12 h after treatment, group 2 is 24 h after treatment, color scale from 1.28e8 µW/cm^2^ to 3.80e8 µW/cm^2^. Orange under cadmium stress ck is the control, group 1 is 100 µmol/L CdCl_2_, group 2 is 200 µmol/L CdCl_2_, color scale from 1.45e8 µW/cm^2^ to 1.35e9 µW/cm^2^. Littleleaf boxwood under drought stress ck is the control, group 1 is 10% PEG6000, group 2 is 20% PEG6000, color scale from 1.70e8 µW/cm^2^ to 2.18e9 µW/cm^2^. **(b)** Relative fluorescence intensity was calculated for each species under different stress levels. For Bottle gourd, a 2*9 cm ROI was selected to compute fluorescence. For Tomato, a 2*4 cm ROI was selected to compute fluorescence. For Orange, a 10*10 cm ROI was selected to compute fluorescence. For Littleleaf boxwood, a 12*12 cm ROI was selected to compute fluorescence. Relative fluorescence intensity values are expressed as mean ± standard deviation, based on three independent replicates.

As shown in **Figure 5a**, in Bottle gourd, Tomato, Orange, and Littleleaf boxwood, low fluorescence was observed under no-stress conditions in the ck group, indicating that the fluorescent probe LWS detected endogenous H_2_O_2_ involved in maintaining normal growth and development. When these four species were subjected to different stress treatments, relative fluorescence intensity increased with increasing stress severity. As shown by statistical analysis of relative fluorescence intensity values in **Figure 5b**, for each species, pairwise comparisons among ck, group 1, and group 2 showed significant differences, and changes in relative fluorescence intensity were positively correlated with stress severity.

#### 3.3.3 Detection of grafting-induced in plant H_2_O_2_ under non-controlled conditions using the fluorescent probe LWS

**Figure 6:**
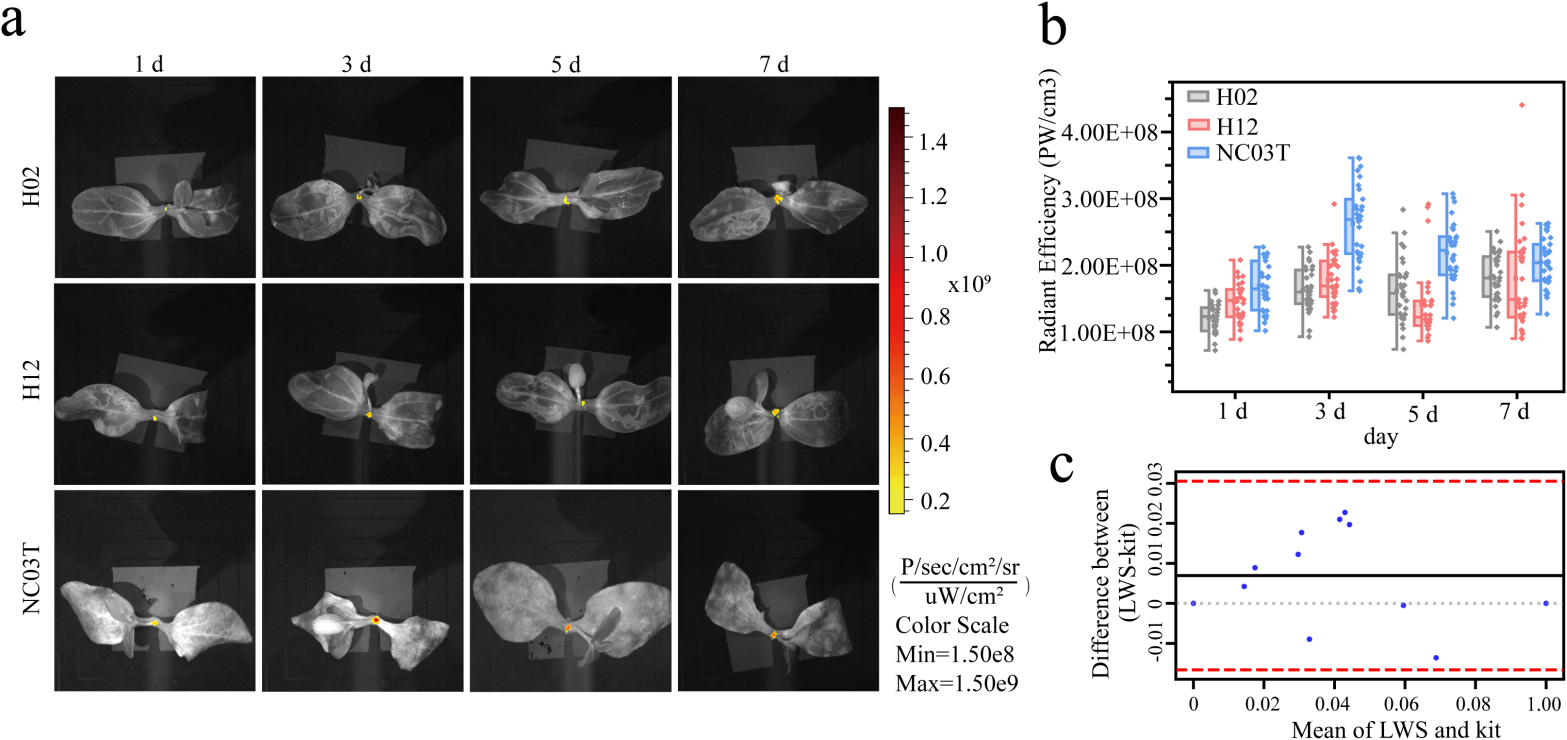
**(a)** Stem imaging of bottle gourd rootstocks H02, H12, and pumpkin rootstock NC03T at days 1, 3, 5, and 7 after grafting, color scale from 1.50E8 µW/cm^2^ to 1.50E9 µW/cm^2^. **(b)** Relative fluorescence intensity calculated from the selected 0.3*0.4 cm ROI area in the stem at different time points for different rootstocks, with a total of 30 samples per time point from three independent experiments. **(c)** Bland-Altman plot showing the agreement between the LWS fluorescent probe method and the commercial kit assay, with the red dashed lines representing the 95% limits of agreement and the black solid line representing the mean difference.

To validate the stem imaging performance of the fluorescent probe LWS and its ability to detect in plant H_2_O_2_ under non-controlled conditions, we combined it with grafting. As shown in **Figure 6a**, the grafted seedlings with the bottle gourd variety H02 as the rootstock exhibited a continuous increase in relative fluorescence intensity at the graft union as the grafting time progressed. For the grafted seedlings with the bottle gourd variety H12 as the rootstock, the relative fluorescence intensity on the third day was stronger than on the first day, while the relative fluorescence intensity on the fifth day was weaker than on the third day, and on the seventh day, the intensity increased again. Statistical analysis was performed on the relative fluorescence intensity produced at the graft union of samples with different rootstocks at different time points. (**Figure 6b**; **Table 1**). Between days 1 and 7, the relative fluorescence intensity of H_2_O_2_ in the grafted seedlings of the H02 variety exhibited more stable changes compared to those of the H12 variety. Subsequently, we calculated the grafting survival rates of the two cultivars, which were 94% for H02 and 64% for H12.

**Table 1:** Statistical table of relative fluorescence intensity at the graft union in different varieties.

Variety	Day	Mean value (p/s)	SD (p/s)	Median (p/s)
H02	1d	1.21E+08	2.18E+07	1.23E+08
H12	1d	1.45E+08	2.87E+07	1.47E+08
NC03T	1d	1.68E+08	3.68E+07	1.64E+08
H02	3d	1.63E+08	3.27E+07	1.60E+08
H12	3d	1.78E+08	3.76E+07	1.69E+08
NC03T	3d	2.62E+08	5.76E+07	2.69E+08
H02	5d	1.62E+08	4.81E+07	1.58E+08
H12	5d	1.41E+08	5.26E+07	1.22E+08
NC03T	5d	2.19E+08	4.67E+07	2.22E+08
H02	7d	1.82E+08	3.63E+07	1.81E+08
H12	7d	1.80E+08	7.83E+07	1.49E+08
NC03T	7d	2.05E+08	3.62E+07	2.04E+08

To verify that our fluorescent probe LWS can also be used in the stem of other plant, we used the pumpkin variety NC03T as the rootstock and the same melon (*Cucumis melo*) variety as the scion to monitor H_2_O_2_ dynamics over days 1-7. In the grafted seedlings with pumpkin as the rootstock, the average relative fluorescence intensity on day 1 was 1.68E + 08 p/s, which increased to a peak of 2.62E + 08 p/s on day 3, and then gradually decreased to 2.05E + 08 p/s. The mean values and standard deviations recorded from days 1 to 7 reflected variations in relative fluorescence intensity and fluctuations in signal levels over time. In addition, we employed a widely used commercial hydrogen peroxide assay kit to measure H_2_O_2_ levels in grafted seedlings subjected to the same treatments, and evaluated the agreement between our method and the commercial kit using Pearson’s correlation coefficient and Bland-Altman analysis. (Millard et al., 2018; Munawar et al., 2019; Wecking et al., 2020) Pearson correlation analysis showed r = 0.999 (p < 0.001), with all data points lying close to the regression line (**Supplementary Figure 3**). Bland-Altman analysis further indicated a very small bias and narrow 95% limits of agreement, with all observations falling within these limits, demonstrating good agreement between our method and the commercial kit and supporting the practical applicability of the LWS fluorescent probe (**Figure 6c**).

## 4 Discussion

In this study, spectroscopy and theoretical analysis confirmed the activation mechanism behind the probe LWS. The probe LWS contains a rhodamine moiety with a conjugated electron system that enables efficient light absorption. However, the presence of a pentafluorobenzenesulfonyl group, which possesses strong electron-withdrawing properties, acts as a quenching group that reduces the fluorescence of the rhodamine core (Chen and Fang, 2023; Liu et al., 2023). Upon the addition of H_2_O_2_, H_2_O_2_ mediates the hydrolysis of the sulfonate, cleaving the pentafluorobenzenesulfonyl group from the rhodamine fluorophore. This cleavage eliminates the quenching effect of the pentafluorobenzenesulfonyl group, allowing the rhodamine fluorophore to revert to its native form. In this restored state, it exhibits a higher molar absorptivity in the ultraviolet region, resulting in an increase in UV absorption. Moreover, the removal of the quenching group restores the fluorophore to a more efficient fluorescent state, thereby enhancing both its absorption and emission properties. This enhancement is further supported by changes in electron cloud distribution and the LUMO-HOMO energy gap before and after the reaction of probe LWS with H_2_O_2_. Due to the strong electron-withdrawing effect of the pentafluorobenzenesulfonyl group in LWS, the π-electron cloud in the LUMO orbital is primarily localized on this group. Upon cleavage of the pentafluorobenzenesulfonyl group, the electron cloud becomes delocalized across the entire fluorophore scaffold. Additionally, the energy gap between the ground state and the excited state in the reaction product LWS-1 is smaller than that of the original probe LWS, allowing for more efficient excitation of electrons and resulting in enhanced fluorescence intensity.

Upon exposure to stress, plants activate a complex network of defense and adaptive responses, initiated by the rapid accumulation of reactive oxygen species as the primary signaling event. Through multi-layered regulatory mechanisms, this network subsequently coordinates key processes, including phytohormone signaling, cell wall remodeling, maintenance of redox homeostasis, and the activation of MAPK cascades, ultimately facilitating the perception, transduction, and response to environmental pressures. As a core signaling molecule, H_2_O_2_ not only triggers the reprogramming of stress-related gene expression by modulating redox states and activating MAPK cascades, but also forms a crucial “ROS-Ca^2+^ crosstalk” hub with second messengers like Ca^2+^ to regulate key enzyme activities. (Li et al., 2022b; Sood, 2025) This signaling module is intricately coupled with phytohormone networks, for instance, H_2_O_2_ and abscisic acid mutually amplify each other during stomatal regulation to promote closure and minimize water loss, while concurrently engaging in crosstalk with defense hormones such as jasmonic acid, salicylic acid, and ethylene. (Bharath et al., 2021) Furthermore, ROS accumulation activates both enzymatic and non-enzymatic antioxidant systems, which can partially mitigate the toxicity of the Fenton reaction by regulating interactions with transition metals like iron. (Dumanović et al., 2021) Subsequently, these biochemical and hormonal signals are manifested at the cellular structural level. In the apoplast, H_2_O_2_ is utilized by cell wall peroxidases to catalyze the oxidative cross-linking of phenolic compounds and hydroxyproline-rich glycoproteins, thereby enhancing cell wall rigidity and forming a physical barrier against pathogen invasion. (Mishler-Elmore et al., 2021; Ma, 2024) Collectively, these studies demonstrate that plant stress responses induce highly dynamic increases in the concentrations of a multitude of substances. These changes in non-H_2_O_2_ species increase the challenge of specifically recognizing H_2_O_2_ by fluorescent probes; therefore, in the design of probe LWS, a pentafluorobenzenesulfonyl group was introduced as the recognition unit. Due to its strong electronegativity, this group significantly enhances the probe’s reactivity toward H_2_O_2_, enabling a highly selective response. This design enables the fluorescent probe to retain high specificity for H_2_O_2_ even within complex biological systems (Qi et al., 2025). In our results, probe LWS exhibited excellent selectivity for H_2_O_2_ and was not affected by abundant endogenous substances commonly coexisting with H_2_O_2_ in plants. These findings demonstrate that probe LWS retains its ability to recognize H_2_O_2_ in complex biological contexts, exhibiting excellent bioapplicability and high specificity.

Endogenous H_2_O_2_ accumulation in plants increases with the severity of stress, especially at early stages when H_2_O_2_ levels are positively correlated with stress intensity (Sachdev et al., 2021). In our results, across the four species tested under salt, drought, cadmium, and waterlogging stresses, fluorescence values rose significantly with increasing stress shortly after stress onset and exhibited a positive correlation with stress severity, consistent with the typical response pattern of plants under stress. This indicates that the fluorescent probe LWS can be used to detect the concentration of in plant H_2_O_2_ across different plants under various types of stress when stress intensity is experimentally controlled, demonstrating broad applicability.

Grafting is the process of joining two different plants to enhance crop disease resistance and quality, and it represents a form of plant tissue repair. During this process, plants with different graft compatibilities produce varying concentrations of H_2_O_2_ at the graft union site. Previous studies have indicated that cucurbit crops exhibit significant physiological and biochemical responses within the first 7 days following grafting, during which reactive oxygen species levels, including hydrogen peroxide, display marked differences. (Miao et al., 2021; Wang et al., 2025) Therefore, setting the measurement window for hydrogen peroxide content within 7 days after grafting is sufficient to effectively capture oxidative stress responses during the early stage of grafting, and also allows verification of whether our method can detect hydrogen peroxide under complex and variable environmental conditions. It has been reported that oxidative bursts are commonly triggered during the initial phase of graft healing, whereas the antioxidant system is relatively compromised in incompatible grafts, thereby leading to greater H_2_O_2_ accumulation after wound healing than in compatible grafts (Aloni et al., 2008; Babar et al., 2023). Subsequently, callus formation likely began in the compatible grafts, and the associated increases in cell division, differentiation, and metabolic activity could have led to elevated H_2_O_2_ production (Irisarri et al., 2015; Carmach et al., 2023). In incompatible grafts, the antioxidant system is relatively weak and fails to scavenge H_2_O_2_ in a timely manner, meanwhile, delayed callus differentiation further contributes to the continued accumulation of H_2_O_2_ (Pina et al., 2012; Sofo et al., 2015; Babar et al., 2023). On day 5, the formation of active callus tissue and a robust antioxidant system in the compatible graft combination maintained a relative balance, resulting in no marked change in H_2_O_2_ accumulation. (Miao et al., 2019; He et al., 2025) In contrast, the callus tissue of the incompatible graft seedlings showed low cellular activity, allowing the antioxidant system to gradually remove the previously accumulated H_2_O_2_. (Thomas et al., 2024) By day 7 after grafting, vascular reconnection began to occur, which increased cellular activity at the graft union. As a result, the rate of H_2_O_2_ production exceeded its scavenging rate, leading to renewed H_2_O_2_ accumulation. (Feng et al., 2024) We observed that the fluorescence changes in **Figure 6a** were consistent with previous studies describing distinct stages during graft union healing. To further confirm that our method correctly captured the oxidative status at different stages in grafted seedlings, we analyzed the overall distribution of relative fluorescence intensity across the tested population and found that from day 1 to day 7 the mean relative fluorescence intensity of the compatible graft combination (H02) remained close to the median, indicating more concentrated and stable data. In contrast, for the incompatible graft (H12), although the mean on day 1 was close to the median, the means from days 3 to 7 deviated significantly from the median. Overall, the standard deviation of relative fluorescence intensity in the compatible rootstock H02 was smaller than that in the incompatible rootstock H12. Previous studies have suggested that, since H_2_O_2_ functions as a signaling molecule, the stability of its concentration may indicate how well plants coordinate wound responses and graft healing. In compatible grafts, a more efficient antioxidant system may rapidly establish homeostasis and maintain H_2_O_2_ at a controllable level for hormonal coordination during healing. (Rasool et al., 2020; Wang et al., 2024a) Conversely, physiological incompatibility between the rootstock and scion can cause inconsistent stress responses in incompatible grafts, resulting in heterogeneous physiological reactions during the healing process. (Babar et al., 2023; Wang et al., 2024a) These results indicate that the *in vivo* hydrogen peroxide patterns captured by our method are consistent with the trends and fluctuations observed in rootstocks with high versus low graft survival rates, and that our method remains effective for grafted seedlings under complex conditions where stress intensity is not artificially controlled. Whether in experimentally controlled settings or in grafting models that are more complex, challenging, and not artificially controlled, the fluorescent probe LWS exhibits great potential for screening individual plants with differing stress-response characteristics.

Notably, in **Figure 6b**, we observed that a subset of plants still exhibited H_2_O_2_-associated fluorescence intensities far above the population mean at the later stage, whereas another subset hovered below the mean level at the preceding time point. The pronounced individual-level variability within the same treatment group suggests that oxidative signals associated with graft union healing do not occur synchronously across plants. Moreover, the differences in relative fluorescence intensity among individuals within a population imply the importance of pre-screening individuals to reduce noise in biological studies. For physiological or omics studies that rely on conventional bulked sampling strategies, such “asynchronous individuals” are typically pooled indiscriminately into the same experimental group, which not only dilutes signals that are genuinely associated with healing efficiency or graft incompatibility, but may also obscure the expression patterns of key regulatory genes. By using the LWS probe–IVIS non-destructive detection platform established in this study, we are able to prospectively identify, without disturbing plant integrity, those individuals within the population that exhibit abnormally elevated or reduced H_2_O_2_ levels and to incorporate them into targeted follow-up experiments. More importantly, by imposing a mild stress on seedlings before field planting and then using our method to rapidly identify and select individuals with superior early-stage stress tolerance, pre-planting decisions can be greatly streamlined, resources wasted on poorly adapted plants can be minimized, and the overall uniformity of field stands as well as the consistency of crop performance can be improved.

Commercial H_2_O_2_ assay kits are widely used for the quantitative determination of hydrogen peroxide in plants. The hydrogen peroxide assay kit used in this study detects H_2_O_2_ based on the formation of a yellow peroxytitanium complex produced by the reaction between H_2_O_2_ and titanium sulfate, which shows a characteristic absorbance at 415 nm. The limit of detection (LOD) of the commercial kit is 0.002 μmol/L, which is lower than the 0.645 μmol/L of our method, enabling it to capture lower concentrations of hydrogen peroxide. However, both methods are capable of detecting the physiological values required to distinguish between stressed and unstressed plants. Regarding the linear range, the commercial kit operates between 0.0097 and 1.5 μmol/L. When this limited linear range is used to compare plants under severe oxidative stress conditions, reactive oxygen species accumulate significantly and H_2_O_2_ concentrations may exceed 1.5 μmol/L. This situation can easily lead to inaccurate comparisons of the oxidative status among highly stressed plants. Therefore, while the commercial kit is designed to detect trace amounts of hydrogen peroxide, the LWS probe retains sufficient sensitivity to detect biologically meaningful H_2_O_2_ elevations induced by stress, despite having a higher detection limit. Consequently, our method strikes an optimal balance between analytical sensitivity and the capacity to differentiate plants with varying oxidative states. While the commercial kit allows the quantitative determination of H_2_O_2_ concentration, its workflow requires grinding plant tissues in an ice-water bath, which may cause H_2_O_2_ to degrade before reacting with the kit reagents. By contrast, our method preserves intact tissues, reducing the possibility of H_2_O_2_ degradation. Notably, the commercial kit destructively disrupts plant tissues, preventing the same samples from being used for subsequent studies. Although our method provides only semi-quantitative readouts of plant oxidative status, It lays the groundwork for subsequent investigations by enabling subsequent analyses on the same specimens. As shown in **Supplementary Table 1**, some previously reported sensors exhibit a wider linear range, while others achieve lower detection limits. (Bukhamsin et al., 2022; Park et al., 2023; Perdomo et al., 2023; Zhao et al., 2023; Li et al., 2024; Pan et al., 2024; Wang et al., 2024b; Zhang et al., 2025) However, the detection methods proposed in these studies generally involve some degree of physical injury to the plants; even when such damage is minimal, it may still disturb the individual plants and thereby affect the accuracy of subsequent experiments. Although our fluorescent probe is not the most outstanding in terms of analytical performance, it shows good applicability in plants, as demonstrated in **Figures 5** and **6**, which indicate that it is sufficient for screening individual plants. Importantly, the combination of this probe with our proposed detection method not only enables the identification of hydrogen peroxide, but also allows for the stratification of plants based on their oxidative status, thereby improving sample uniformity for downstream biological studies. Additionally, some non-destructive detection techniques based on spectroscopy or image recognition can significantly improve recognition accuracy when combined with machine learning algorithms, the phenotypic symptoms on which they rely often lag behind stress-induced physiological changes (Ji et al., 2025; Tuccio et al., 2025; Wu et al., 2025). Therefore, these approaches are more suitable for plant stress monitoring at the population scale or over large areas, rather than for precise early identification at the single-plant level. Consequently, in the early detection of plant stress, the timeliness of these sensors still remains clearly inferior to that of small-molecule fluorescent probes that undergo specific chemical reactions with defined physiological indicators. Spectroscopic evidence demonstrated that, in comparison with other small-molecule fluorescent probes, probe LWS provides H_2_O_2_ concentration readouts that approximately reflect the levels of H_2_O_2_ accumulated under stress conditions and enables highly specific detection of H_2_O_2_. In current literature, most fluorescent probe designs focus on rapid detection, typically completing target recognition within seconds or minutes. However, such designs may have limitations when applied to detecting endogenous H_2_O_2_ in plants under stress. When plants are subjected to stress, the accumulation of H_2_O_2_ within plant bodies varies among different cultivars. This differential accumulation modulates the responses of various plant hormones to stress, ultimately leading to distinct physiological outcomes in plant stress tolerance (Kamruzzaman et al., 2022) However, during the application of fluorescent probes in plants, factors such as environmental fluctuations, chemical treatments, and changes in endogenous enzyme activity or hormone levels may induce transient H_2_O_2_ peaks within plant bodies. Although plants possess efficient antioxidant systems that can rapidly eliminate such transient peaks, the H_2_O_2_ detected by rapid-response probes may include these suddenly occurring bursts and therefore cannot accurately reflect the true accumulation level of H_2_O_2_ induced by stress. Therefore, by controlling the reaction time, the LWS probe, while still enabling early detection, yields H_2_O_2_ levels that more closely approximate the accumulation concentration produced in plants under stress.

However, it should be noted that when the heights of the tested sites are not consistent, additional tools, such as the glass tubes used in this study, should be used to control the imaging height to ensure consistency in optical path length and excitation or emission efficiency, thereby eliminating noise caused by positional differences. In addition, because each imaging run of the IVIS *in vivo* imaging system requires a certain amount of time, it is not feasible to process a large batch of plants at once, otherwise differences in sample handling time will be introduced and result in technical noise. When measurements are performed using a standardized operating procedure, the standard deviation of the CK group in **Figure 5b** is smaller than that of the treatment groups, indicating that technical noise is effectively reduced and allowing the biological variation among individual plants to be observed. Because individual plants respond differently to stress, the treatment groups all show larger standard deviations. **Figure 6b** further demonstrates this, as incompatible grafted seedlings show a larger standard deviation. Due to the limited imaging chamber of the IVIS Lumina LT *in vivo* imaging system used in this study, this method is suitable for small plants but cannot accommodate large plants. Therefore, attention should be paid to the size of the target tissue to be measured. The physical limitation of tissue penetration depth remains a major challenge for small-molecule fluorescent probes. This limitation arises from the optical properties of biological tissues, such as light scattering and absorption, which cause the signal detected by IVIS *in vivo* imaging to mainly originate from superficial layers and thus fail to reflect H_2_O_2_ levels in deep tissues. (Hériché et al., 2022; Refaat et al., 2022) Accordingly, this method is limited to screening plants based on H_2_O_2_ levels generated in superficial tissues. A common challenge for the entry of exogenous substances into plants is that different abiotic stress types induce different degrees of stomatal closure and tissue permeability, thereby affecting probe penetration and in planta enrichment. (Li et al., 2022a; Yang et al., 2023) This reduces direct comparability across different stress types and restricts the method to comparing oxidative status only among plants subjected to the same type of stress, while limitations remain for comparisons between different stress types. In addition, consistent incubation time and washing steps are required to control probe enrichment in planta and to reduce interference from residual probe on the surface. Because our probe minimizes interference from transient fluctuations in H_2_O_2_ by controlling the reaction time, it measures the accumulated H_2_O_2_ level within the incubation window. Compared to other rapid detection methods, by integrating the signal over the incubation period, this method effectively filters out transient H_2_O_2_ fluctuations caused by minor environmental perturbations, chemical treatments, or changes in enzyme activity, thereby capturing the steady-state accumulation of H_2_O_2_. Since the steady-state accumulation of H_2_O_2_ inherently serves as a crucial indicator of biological phenotypes, this approach provides more reliable data for evaluating long-term physiological stress, making it particularly suitable for studying chronic or prolonged stress states with enduring effects. It should be noted, however, that this also defines its application boundaries. When the research conditions or objectives require high-temporal-resolution and highly dynamic monitoring of stress responses, or necessitate pinpointing the exact temporal nodes of H_2_O_2_ signal transduction, this method fails to capture these instantaneous changes in hydrogen peroxide concentrations.

## 5 Conclusion

We have developed a novel fluorescent probe for H_2_O_2_ detection with excellent sensitivity and selectivity. This probe avoids transient H_2_O_2_ bursts caused by environmental stimuli or metabolic fluctuations in plants, thereby providing more reliable steady-state H_2_O_2_ measurements. Additionally, the probe functions effectively under physiological pH conditions found within plant tissues and exhibits no detrimental effects on plant growth and development at concentrations below 50 µmol/L. By applying the fluorescent probe LWS to different plants under various stress treatments, we observed that as stress severity increased, the relative fluorescence intensity detected by the IVIS Lumina LT *in vivo* imaging system rose with increasing oxidative stress, consistent with the typical response pattern of plants under stress. Similarly, when the fluorescent probe was applied to graft unions of seedlings from different cultivar combinations, statistical analyses showed relative fluorescence intensity changes consistent with earlier reports, and the measurements were consistent with the results obtained from a commercial kit. These results indicate that combining our H_2_O_2_ fluorescent probe with the IVIS Lumina LT imaging system and quantitative analysis using Living Image software enables precise quantification of relative fluorescence intensity across different plant tissues under various stresses. This method allows non-destructive screening of plants with similar physiological states, preserving plant integrity. Compared to traditional chemical detection methods, our approach maintains the integrity of plant tissues, facilitating subsequent omics and phenotypic analyses. In addition, before field cultivation, our method allows the selection of more stress-tolerant plant individuals, thereby ensuring crop yields under climate change conditions. In summary, our method provides a simple, effective, and non-destructive approach for measuring H_2_O_2_ concentrations in plants, laying a technical foundation for dissecting the regulatory networks underlying plant stress responses.

## Data Availability

The original contributions presented in the study are included in the article/**Supplementary Material**. Further inquiries can be directed to the corresponding author.
