# Supplementary material for: A non-destructive plant screening method for improving sample uniformity in horticultural crops based on a hydrogen peroxide fluorescent probe
Source: Front Plant Sci. 2026 Mar 9;17:1767323. doi: 10.3389/fpls.2026.1767323 (PMC13006615; doi:10.3389/fpls.2026.1767323)
Supplement: Supplementary file 1 [file DataSheet1.docx]

Supplementary Material

# Mass spectrometry (MS) analysis of the incubated samples

The concentration of the probe stock solution was prepared at 10 mmol/L by dissolving it in chromatographic-grade dimethyl sulfoxide (DMSO), while the concentration of the H₂O₂ stock solution was set at 100 μmol/L. The reaction system employed a methanol/phosphate buffer mixture (v/v = 6:4) as the solvent. A volume of 50 μL of the probe stock solution and an appropriate amount of the H₂O₂ stock solution were mixed and diluted with the reaction solvent to a final volume of 3 mL. The reaction mixture was incubated in a 37°C water bath for 1 hour, followed by drying using a rotary evaporator. The dried samples were then dissolved in mass spectrometry-grade methanol, filtered through a 0.22 μm microporous membrane, and subsequently subjected to mass spectrometric analysis.

# Theoretical calculations and analysis

Using the Gaussian 16 software and based on density functional theory (DFT), the spatial chemical structures, electron cloud distributions, and electronic transition energy levels of the probe LWS and the final product LWS-1 were calculated. The B3LYP/6-31G(d,p) basis set was employed to perform ground-state energy optimizations (lowest-energy conformations) for both molecules.

# Calculation of the limit of detection (LOD)

The limit of detection (LOD) of the probe was calculated using the following formula:

$$\sigma=\sqrt{\frac{1}{N}\Sigma_{i-1}^{N}\left( x_{i}-\mu\right)^{2}}$$

$$LOD=\frac{3\sigma}{k}$$

In this formula, σ represents the standard deviation of the fluorescence intensity of the pure probe measured in a quartz cuvette during the fluorescence detection experiments. k denotes the slope of the fluorescence titration curve obtained through linear calibration fitting.

# Supplementary Figures


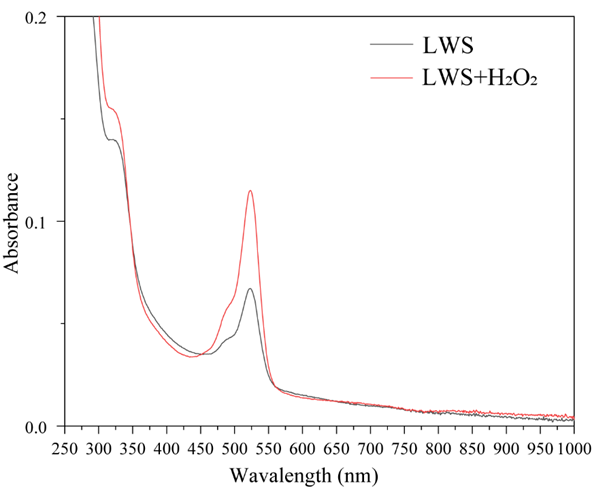


**Supplementary Figure 1.** UV absorption spectrum of probe LWS toward H₂O₂


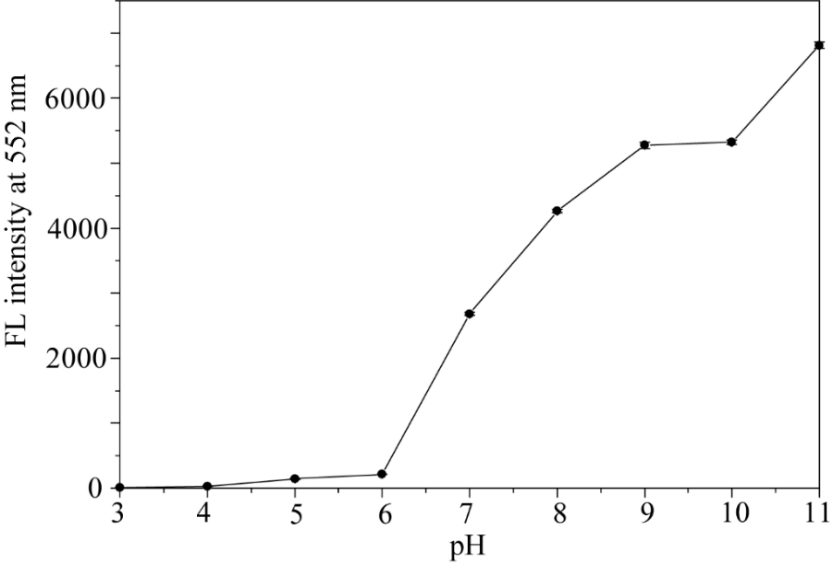


**Supplementary Figure 2.** Fluorescence spectra of probe LWS after reaction with H₂O₂ under different pH conditions

**Supplementary Figure 3.** Pearson correlation analysis of H₂O₂ measurements obtained using the LWS fluorescent probe method and a commercial assay kit in grafted seedlings (r = 0.999, p < 0.001).


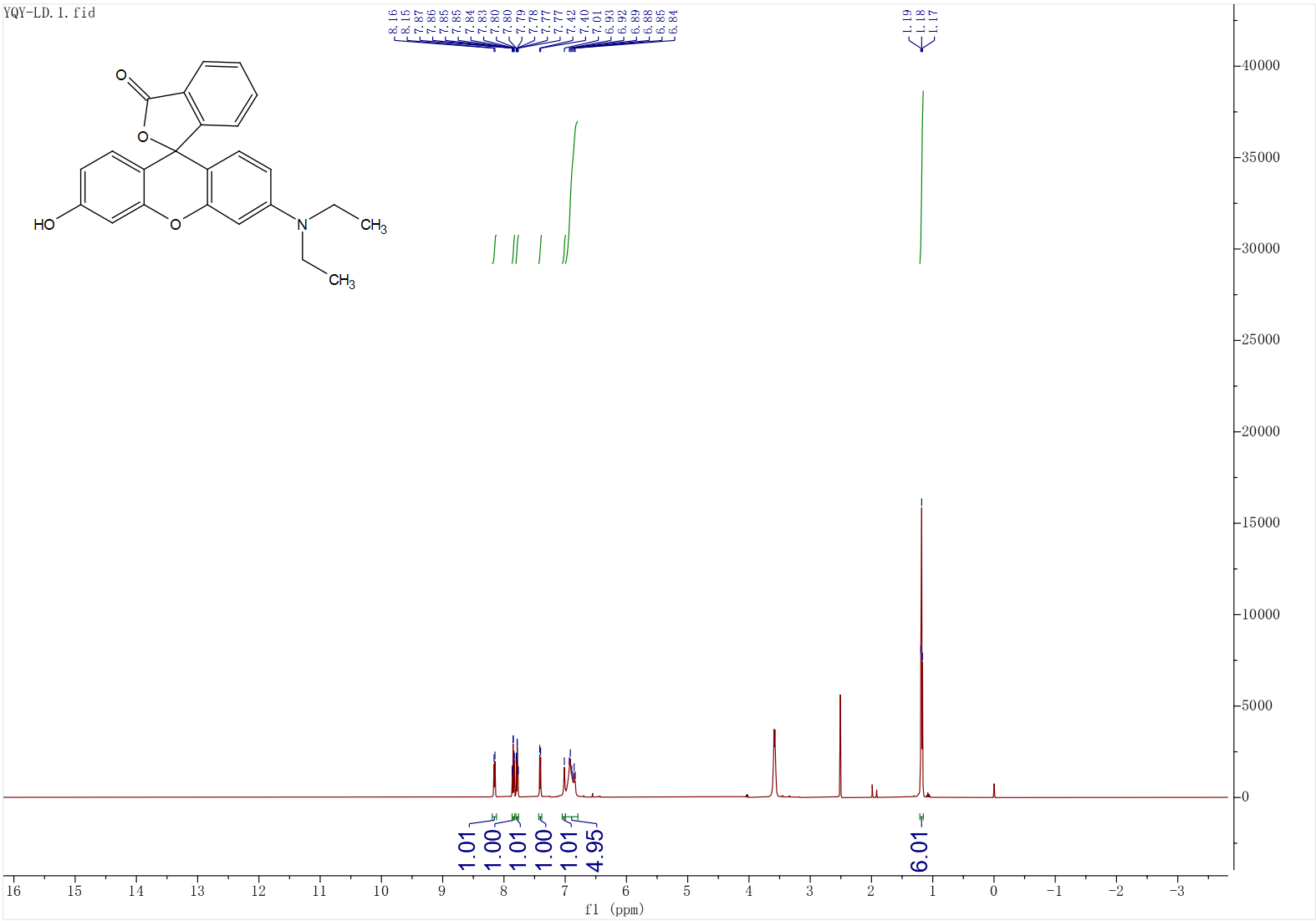


**Supplementary Figure 4.** ¹H NMR spectrum of rhodamine derivative 1 in DMSO


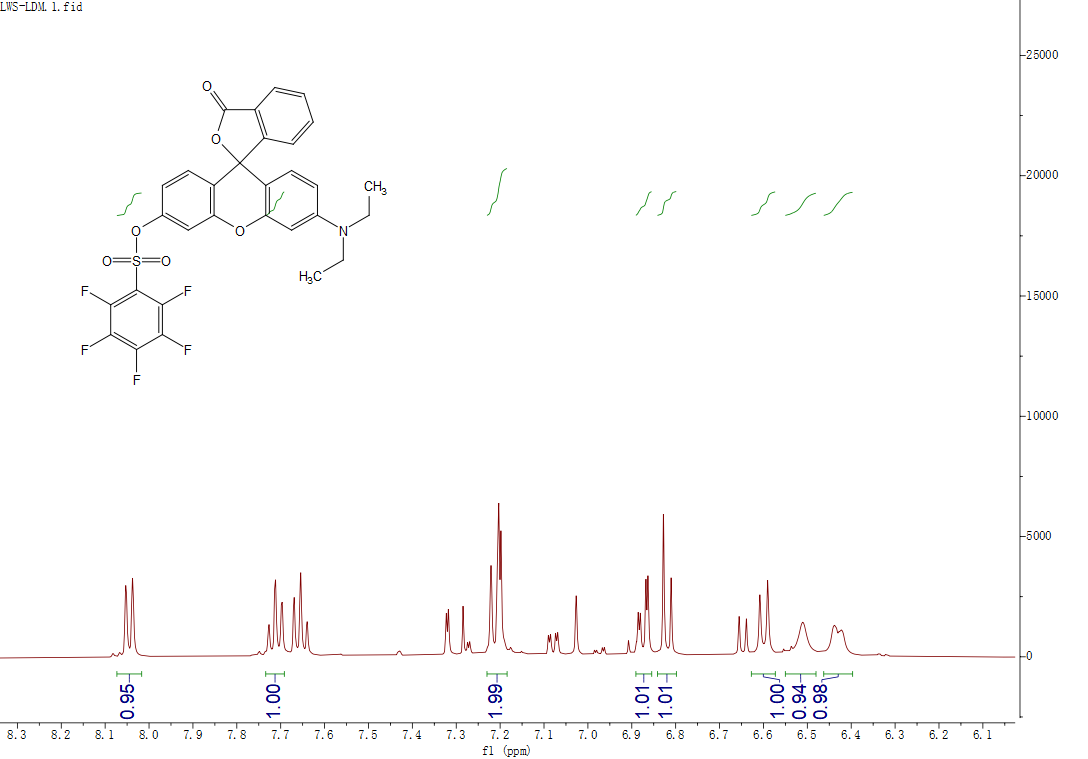


**Supplementary Figure 5.** ¹H NMR spectrum of fluorescent probe LWS in CDCl_3_

**Supplementary Figure 6.** ¹³C NMR spectrum of rhodamine derivative 1 in DMSO


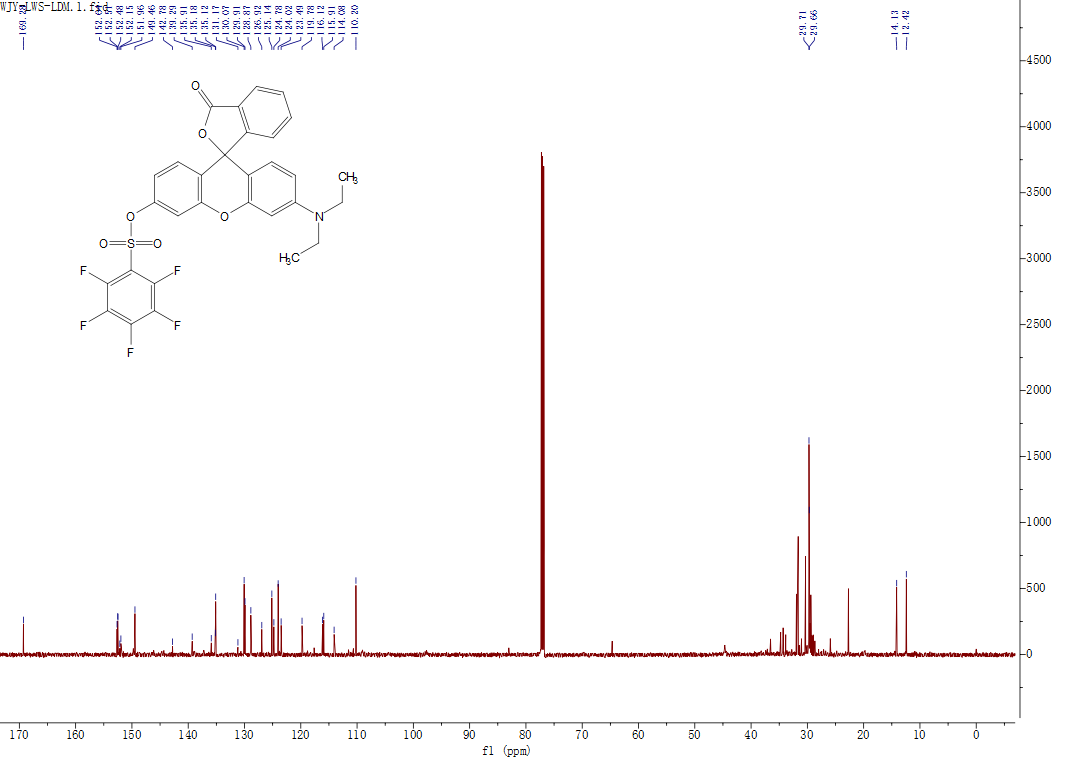


**Supplementary Figure 7.** ¹³C NMR spectrum of fluorescent probe LWS in CDCl_3_


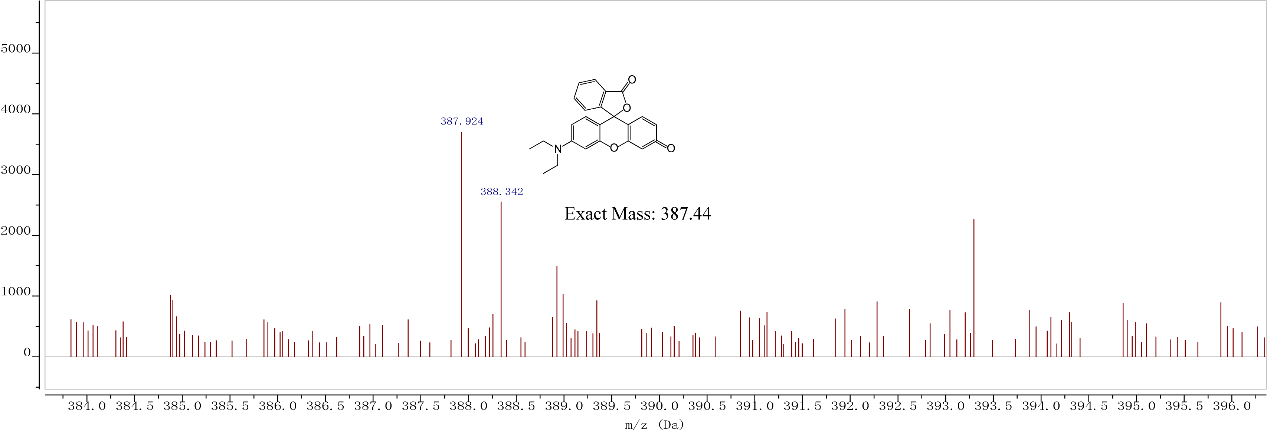


**Supplementary Figure 8.** Mass spectrum of rhodamine derivative 1

**Supplementary Figure 9.** Mass spectrum of fluorescent probe LWS

**Supplementary Table 1.** Summary of the features of the currently available H_2_O_2_ assays

| **Detection method** | **Linear range(μM)** | **LOD(μM)** | **Sampling invasiveness** | **Reference** |
| --- | --- | --- | --- | --- |
| HPLC-DAD | 14.71–2941.17 | 8.82 | Destructive | Park et al., 2023 |
| HPLC-FLD | 0.15–11.76 | 0.03 | Destructive | Park et al., 2023 |
| Electrochemistry | 5 ~ 150 | 2.74 | Minimally invasive | Bukhamsin et al., 2022 |
| Electrochemistry | 20-80 | 9.4 | Non-destructive | Perdomo et al., 2023 |
| Colorimetric | 0.05–25 | 0.03 | Minimally invasive | Li et al., 2024 |
| Fluorescence | 0–40 | 0.16 | Ex vivo (destructive) | Pan et al., 2024 |
| Fluorescence | 0–60 | 0.0284 | Ex vivo (destructive) | Wang et al., 2024 |
| Fluorescence | 0–6 | 0.34 | Ex vivo (destructive) | Zhang et al., 2025 |
| Fluorescence | 0.2–42 | 0.05 | Ex vivo (destructive) | Zhao et al., 2023 |
| Fluorescence | 0-100 | 0.645 | Non-destructive | this work |
